# Two coupled chains are simpler than one: field-induced chirality in a frustrated spin ladder

**DOI:** 10.1038/s41598-020-72215-z

**Published:** 2020-09-28

**Authors:** Marek Pikulski, Toni Shiroka, Francesco Casola, Arneil P. Reyes, Philip L. Kuhns, Shuang Wang, Hans-Rudolf Ott, Joël Mesot

**Affiliations:** 1 Laboratory for Solid State Physics, ETH Zürich, 8093 Zürich, Switzerland; 2 Paul Scherrer Institut, Villigen PSI, 5232 Villigen, Switzerland; 3 Harvard-Smithsonian Center for Astrophysics, Harvard University, Cambridge, MA 02138 USA; 4 National High Magnetic Field Laboratory, Florida State University, Tallahassee, FL 32310 USA; 5 Laboratory for Quantum Magnetism, Ecole Polytechnique Fédérale de Lausanne, 1015 Lausanne, Switzerland

**Keywords:** Electronic properties and materials, Magnetic properties and materials

## Abstract

Although the frustrated (zigzag) spin chain is the Drosophila of frustrated magnetism, our understanding of a pair of coupled zigzag chains (frustrated spin ladder) in a magnetic field is still lacking. We address this problem through nuclear magnetic resonance (NMR) experiments on BiCu$_2$PO$_6$ in magnetic fields up to 45 T, revealing a field-induced spiral magnetic structure. Conjointly, we present advanced numerical calculations showing that even a moderate rung coupling dramatically simplifies the phase diagram below half-saturation magnetization by stabilizing a field-induced chiral phase. Surprisingly for a one-dimensional model, this phase and its response to Dzyaloshinskii-Moriya (DM) interactions adhere to classical expectations. While explaining the behavior at the highest accessible magnetic fields, our results imply a different origin for the solitonic phases occurring at lower fields in BiCu$_2$PO$_6$. An exciting possibility is that the known, DM-mediated coupling between chirality and crystal lattice may give rise to a new kind of spin-Peierls instability.

## Introduction

**Figure 1:**
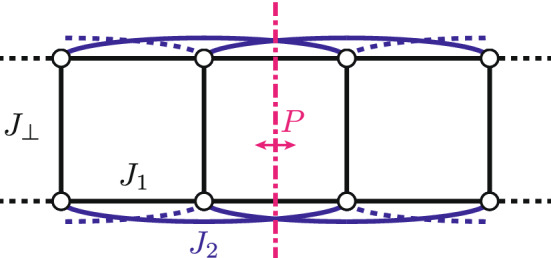
Frustrated spin ladder. A finite segment of the infinitely-extended frustrated spin-ladder model. Vertices correspond to spin-${1}\big /{2}$ magnetic moments, while edges represent the relevant exchange interactions $J_1$, $J_2$, and $J_\perp$. The system exhibits a reflection symmetry denoted as *P*.

Despite previous studies^1–6^, the effects of external magnetic fields on materials in which antiferromagnetic (Heisenberg) exchange interactions between spin-${1}\big /{2}$ moments form quasi one-dimensional frustrated spin ladders (Fig. 1) are not fully understood. The frustrated ladder model^7^ encompasses the unfrustrated spin ladder^8^ and the spin chain with frustrating next-nearest neighbor (NNN) interactions (zigzag chain)^9,10^, as limiting cases for $J_2 = 0$ and $J_{\perp } = 0$, respectively^11^. Given a sufficiently-strong frustration ratio $J_2/J_1$ in the latter case, both the aforementioned systems adopt a spin-singlet ground state with a spin gap and short-range spin correlations only^8,12,13^—two hallmarks of quantum spin liquids (QSLs)^14^.

Closing the gap by applying a magnetic field typically induces magnetic order in such systems^15^. For example, a field-induced Bose-Einstein condensation (BEC) of mobile spin-triplet excitations (triplons) gives rise to antiferromagnetic (AFM) order in the unfrustrated spin ladder with dominant rung exchange $J_\perp$^16^. On the other hand, the frustration of the zigzag chain entails dimerization^17^ and incommensurate, spiral-like spin correlations^18,19^, resulting in more complicated field-induced phases^20^. In particular, the zigzag chain supports field-induced chiral order^20–23^, which can be described as a condensation of magnetic excitations with incommensurate wavevector^24^. This order is characterized by translationally-invariant expectation values of the longitudinal component of the chirality operator $\varvec{\kappa }_{ij} = \varvec{S}_i \times \varvec{S}_j$ (cf. Eq. 7 in Ref. 25) quantifying the “twist” of the magnetic structure along a bond (*i*, *j*) between two magnetic sites with spin operators $\varvec{S}_i$ and $\varvec{S}_j$^22^. Note that chiral order only breaks^19^ the discrete reflection symmetry *P* (Fig. 1) and can therefore occur even in the absence of ordered moments^26^.

Intuitively, the ordered state corresponds to a spiral structure with fixed handedness (left- or right-handed), but undefined propagation phase. This is plausible, given that such spiral structures form the ground states of the classical zigzag chain ($S \rightarrow \infty$) and are, indeed, expected to arise also as a field-induced phase of the quantum ($S=1/2$) zigzag chain, if residual interchain couplings permit conventional long-range magnetic order^20^. Such spiral order corresponds to a secondary breaking of the *U*(1) symmetry emerging^24^ from the combination of lattice-translation invariance and incommensurate spin correlations [in infinitely-extended systems this quasi-continuous *U*(1)-symmetry persists even if the continuous *SU*(2) symmetry of the magnetic moments themselves is fully lifted, e.g.,^43^ by an external magnetic field and DM interactions]. Conceptually, this situation is similar to the aforementioned appearance of long-range AFM order following a field-induced BEC of triplons in the unfrustrated spin ladder.

**Figure 2:**
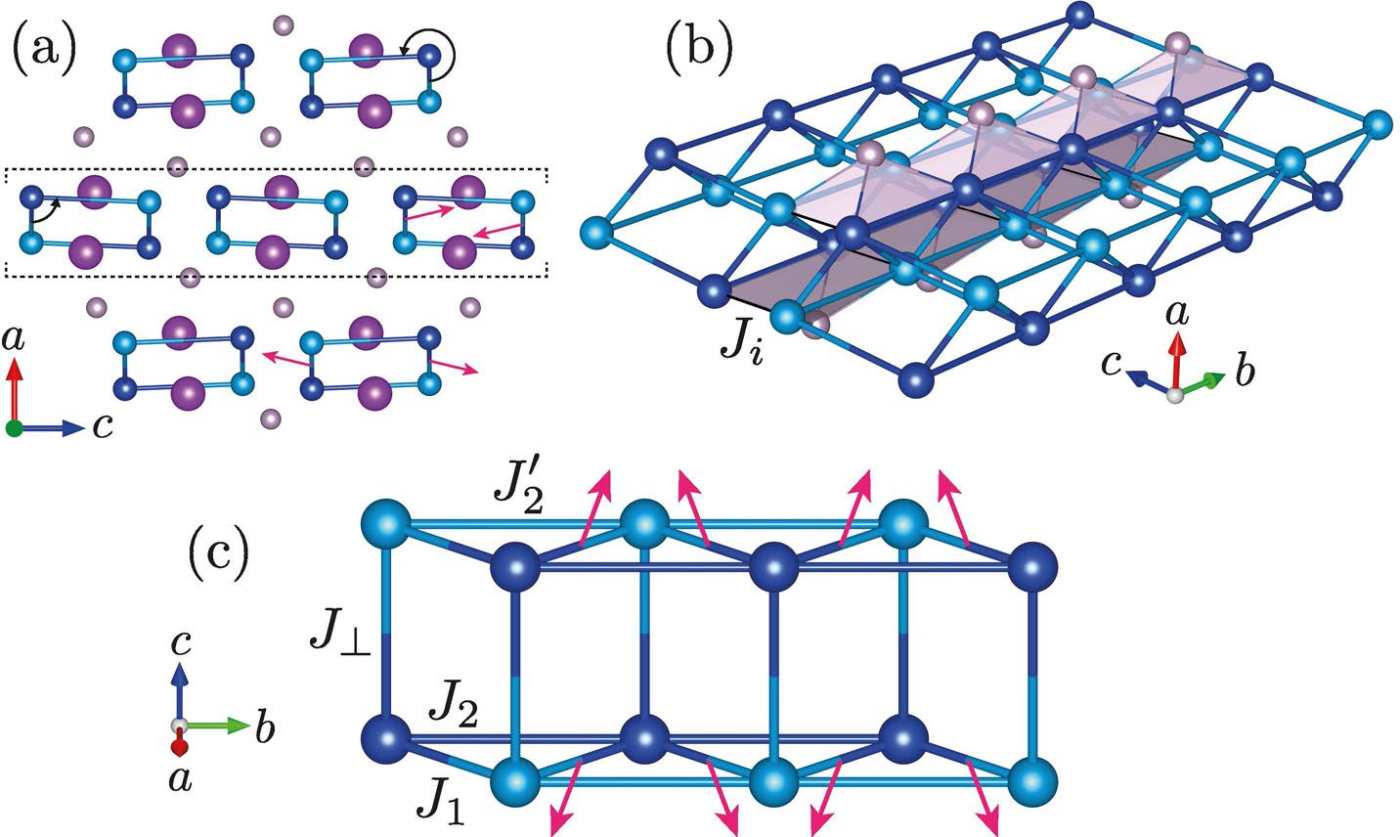
Crystal structure of BiCu$_2$PO$_6$. The positions^27^ of Cu (light and dark blue), P (gray) and Bi (purple) are shown; O sites have been omitted for clarity. Exchange interactions^1^ ($J_1$, $J_2^\prime$, $J_2$, $J_\perp$, and $J_i$) are depicted by blue (intraladder couplings) and black (interladder couplings) lines. (**a**) View along *b*, illustrating the two ladder orientations. Dashed lines mark one ladder layer. (**b**) Two ladder units. Hyperfine couplings^28^ between $^{31}$P nuclei and Cu sites are represented by gray pyramids. (**c**) Single ladder. Magenta arrows in (**a**) and (**c**) illustrate the staggering of the transverse and parallel components, $D_1^{ac}$ and $D_1^b$, of the DM vectors^29–31^ on the nearest-neighbor bonds [projected into *bc* plane in (**c**)]. Illustrations created using VESTA^32^.

The zero-field ground-state phase diagram of the frustrated spin ladder (Fig. 1) essentially interpolates between the unfrustrated spin ladder and the zigzag chain^11^. Specifically, the dimerized character of the zigzag chain is retained for small rung couplings, whereas stronger rung couplings yield a uniform ground state with dominant resonating-valence-bond (RVB) contributions from the rung bonds^7,11^. As in the zigzag chain, sufficient frustration induces incommensurate spin correlations^11^ and chiral order thus becomes possible^2,5,6^.

Our work aims at understanding the intriguing variety of field-induced phases reported previously^5,6,33,34^ for BiCu$_2$PO$_6$^27^, which is believed to be described by the frustrated spin-ladder model depicted in Fig. 2^1,29–31,35–38^. We address this question through both new calculations and new high-field experiments. Moreover, to allow for a meaningful comparison with the real compound, our considerations account for the presence of two inequivalent magnetic sites with corresponding next-nearest neighbor (NNN) couplings ($J_2^\prime$ and $J_2$)^1^, as well as various symmetry-allowed Dzyaloshinskii-Moriya^39,40^ (DM) interactions^29–31^. Previous numerical calculations for BiCu$_2$PO$_6$ revealed the appearance of field-induced chirality for a particular choice of model parameters^5,6^. However, the dependence of the field-induced chiral phase on the exchange couplings and on the DM interactions was not considered in detail. The calculations further seemed to indicate the presence of another magnetic phase at low system magnetizations. On the other hand, similar features had been attributed to convergence problems in previous work on the zigzag chain^41^. In the following, we report the results of *comprehensive numerical calculations* for the frustrated ladder model (Fig. 2c), which clarify that (i) a field-induced chiral phase generally appears for sufficiently strong frustration and rung coupling, and (ii) no additional field-induced phases occur at lower magnetizations. Overall, the field-induced phases occurring below half-saturation magnetization are much more similar to the classical ground state than it is the case for an isolated zigzag chain.

To connect our calculations with experimental observations, we present $^{31}$P nuclear magnetic resonance (NMR) data collected on BiCu$_2$PO$_6$ in high magnetic fields $\varvec{H} \parallel b$. For this orientation, magnetic phase transitions were observed at critical fields $\mu _0\, H_{c1} \simeq 20$ T and $\mu _0\, H_{c2} \simeq 34$ T (for $T \sim 0$ K)^33^. The state above $H_{c1}$ was interpreted as a soliton lattice, and an instability towards chiral order at even higher fields was proposed^5,6^. Our new data are consistent with the latter prediction and we offer new insights by discussing the results in view of our new calculations, as well as other experiments, including measurements of the electric polarization^42^.

**Figure 3:**
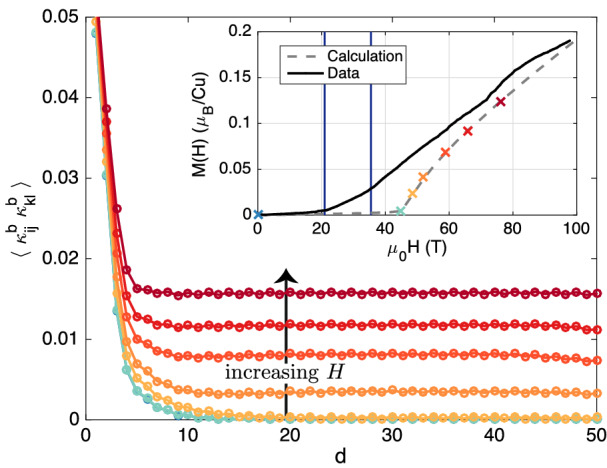
Chiral correlations. Correlations of longitudinal chirality $\kappa _{ij}^b$ on two nearest-neighbor bonds $\langle ij \rangle$ and $\langle kl \rangle$ as a function of the distance *d* along the ladder leg, calculated for different magnetic fields (cf. inset). Inset: Calculated magnetization, along with data reported in Ref. 34. Crosses indicate the fields at which the correlation functions shown in the main panel were evaluated and vertical blue lines correspond to the experimental $H_{c1}$ and $H_{c2}$ values^33^. The continuous evolution of the calculation results with *H* is due to the symmetry-lowering DM interactions (cf., e.g., Ref. 43).

## Results

### Numerical calculations for the frustrated-ladder model

We model the system shown in Fig. 2c using the zero-field spin Hamiltonian^30,31^

1 $$\begin{aligned} {H_0} = \sum _{\langle ij \rangle } \left[ J_{ij}\; \varvec{S}_i \cdot \varvec{S}_j + \varvec{D}_{ij} \cdot \left( \varvec{S}_i \times \varvec{S}_j \right) + \varvec{S}_i \cdot \varvec{\Gamma }_{ij} \cdot \varvec{S}_j \right], \end{aligned}$$

where $\langle ij \rangle$ iterates over pairs of interacting sites *i* and *j*, and $\varvec{D}_{ij}$ denotes the DM vectors. The symmetric tensor $\varvec{\Gamma }_{ij}(\varvec{D}_{ij})$ arises for anisotropic superexchange interactions^44,45^. An external magnetic field introduces an additional Zeeman coupling term, $H_Z = \sum _i \mu _0\,\varvec{H} \cdot \mathrm {g}_i\,\mu _B\,\varvec{S}_i$ (cf., e.g., Ref. 36). We assume that $\varvec{H} \parallel b$ in the experiment, which replaces the two inequivalent g-tensors by scalars^6^. Further details regarding the modeling are described in “Methods”.

Calculated correlation functions of the longitudinal chirality are shown in Fig. 3. The correlations are short ranged until the applied magnetic field is large enough to suppress the spin gap (kink in the calculated magnetization) and become long ranged immediately thereafter. In accordance with an incipient order with spiraling transverse magnetic moments, the asymptotic behavior of the transverse spin correlations concomitantly switches from short-ranged to slowly-decaying.

**Figure 4:**
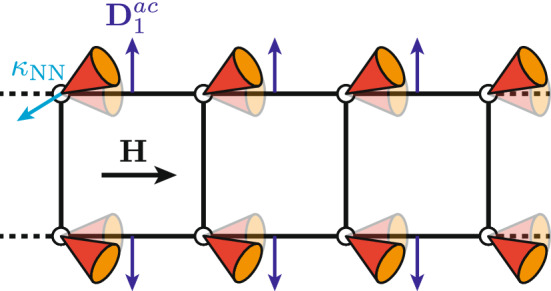
Spiral structure with DM-induced distortions. Schematic illustration of tilt distortion expected due to the transverse DM interaction $\varvec{D}_1^{ac}$ (Fig. 2c). The cones represent canted spiraling magnetic moments with incommensurate propagation along the leg direction and $\varvec{\kappa }_\text {NN}$ denotes the vector chirality on the nearest-neighbor leg bonds. Opaque (transparent) colors are used to depict the situation with (without) DM interactions.

The above behavior is robust against moderate DM interactions (see “Methods”). Since the DM interactions can be written as $\varvec{D}_{ij} \cdot \varvec{\kappa }_{ij}$, it is natural to expect DM-induced twist- and tilt distortions for classical spiral structures (see, e.g., Ref. 46). The latter case is illustrated in Fig. 4. Indeed, we found the correlation functions in the field-induced chiral phase of the ideal one-dimensional quantum system to be altered accordingly in the presence of DM interactions. Although the aforementioned tilt distortion is invoked for the quantitative discussion of the experimental NMR spectra (cf. “Methods”), DM interactions are not essential for a qualitative understanding of the field-induced chiral order. However, they reduce the spin-space symmetry (see, e.g., Ref. 43) and thus allow finite-size systems to exhibit incommensurate field-induced long-range magnetic order in the ground state (see “Methods”).

**Figure 5:**
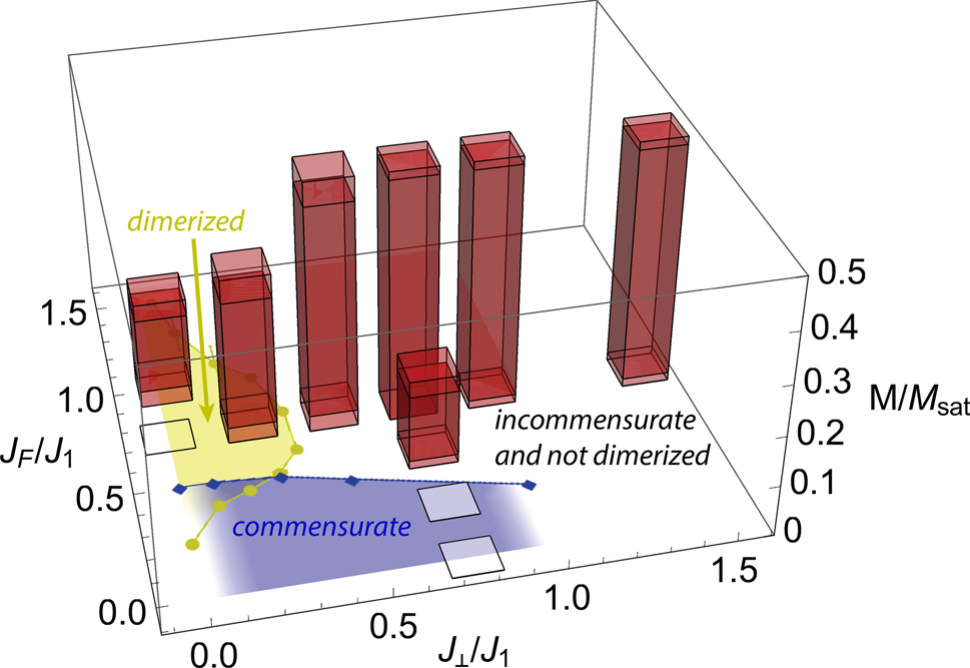
Influence of exchange couplings. Magnetization region occupied by the chiral phase (red bars), as a function of exchange couplings [$J_F = (J_2 + J_2^\prime )/2$, $J_2 / J_2^\prime = 2$ fixed]. The magnetization *M* is normalized to the saturation magnetization $M_\text {sat}$. The end regions of red bars are plotted less opaque to represent uncertainties as applicable. Transparent squares, centered at the corresponding values of $J_F$ and $J_\perp$, indicate the projections of the red bars onto the $M=0$ plane. The zero-field phase boundaries towards the dimerized (light green) and commensurate phases (blue) of the frustrated ladder with $J_2=J_2^\prime$^11^, redrawn using data points provided by the authors of Ref. 11, are included for comparison.

The influence of the exchange interactions, in the absence of DM interactions, is illustrated in Fig. 5. Note that we assumed two inequivalent NNN bonds ($J_2^\prime \ne J_2$) for consistency with our experimental work on BiCu$_2$PO$_6$, which deviates from $J_2^\prime = J_2$ used in Ref. 11. However, besides allowing for different ordered moments on the two magnetic sites, we find no effects of this assumption on the zero-field phase diagram or the field-induced chiral phase mapped in Fig. 5 (see “Methods”).

**Figure 6:**
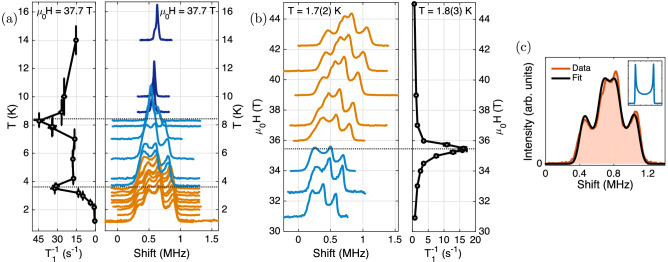
High-field NMR data. (**a**,**b**): $^{31}$P-NMR spectra and relaxation rates $T_1^{-1}$ of BiCu$_2$PO$_6$, as function of (**a**) temperature *T* and (**b**) magnetic field $\mu _0\, H$ ($\varvec{H} \parallel b$). The baseline ordinates of the spectra encode the corresponding temperatures and magnetic fields, respectively. Spectra are normalized to their maximal intensities. Colors distinguish different phases and dotted lines indicate the approximate locations of the peaks in $T_1^{-1}$. (**c**): $^{31}$P-NMR spectrum measured at $\mu _0\, H = 42.2\ \mathrm {T}$ and $T = 1.7\ \mathrm {K}$, along with the fitted line shape. The inset shows a typical double-horn spectrum.

### High-field NMR

Figure 6a,b shows the measured NMR spectra and relaxation rates. The effects of decreasing temperature in high fields (a) and of increasing field at low temperature (b) are similar. The $^{31}$P-NMR spectrum, which reflects the distribution of internal magnetic fields at the P site, first evolves from a single line (dark blue spectra) to a three-peak structure (light blue spectra) reported^5, 6^ earlier (see “Methods”). At even higher fields, a fourth peak develops (orange spectra). These distinct changes are accompanied by peaks in the spin-lattice relaxation rate $T_1^{-1}$, which are indicative of slow magnetic fluctuations. Together with the smooth evolution of the spectra, this confirms^33^ the presence of two second-order phase transitions at temperatures and fields consistent with Ref. 33.

## Discussion

Based on the behavior of the calculated correlation functions (cf. Fig. 3), a field-induced chiral—or, accordingly, spiral—phase is the only phase expected to appear at magnetizations which are of experimental relevance for BiCu$_2$PO$_6$. More generally, Fig. 5 admits the conjecture that a direct field-induced transition from the spin-singlet ground state to a chiral phase occurs for all sets of exchange couplings giving rise to a non-dimerized ground state with incommensurate spin correlations. The latter aspect is consistent with the fact that the breaking of the reflection symmetry *P* requires a ground state with low-symmetry lattice momentum^24^, whereas the former condition suggests that dimerization and chirality are competing phenomena. Comparison with the phase diagram^20^ of the isolated zigzag chain ($J_\perp = 0$) finally shows that the field-induced chiral phase of the frustrated spin ladder is connected to that of the zigzag chain in the space of exchange couplings. Moreover, the rung coupling is found to simplify the phase diagram below half-saturation magnetization by suppressing the various competing phases appearing in the zigzag chain^20^ in favor of a single field-induced chiral phase. Remarkably, due to the similarity^20^ between chiral order and the classically-expected spiral structures revisited in the introduction, the frustrated two-leg ladder thus turns out to be “much more classical” than an individual zigzag chain. This observation is further corroborated by the fact that the DM-induced changes to the correlation functions are consistent with those expected for classical spiral structures. Nevertheless, the quantum nature of the considered model clearly remains relevant, e.g., for explaining the presence of a spin gap with associated field-induced order^15^.

After these general results for the frustrated ladder system, we discuss the high-field NMR spectra obtained in BiCu$_2$PO$_6$ in more detail. Based on the calculations reported in Ref. 6 and this work, a field-induced incommensurate spiral structure—possibly with DM-induced distortions—is expected to appear in BiCu$_2$PO$_6$. With the usual assumption of linear hyperfine couplings, such magnetic structures quite generally give rise to double-horn spectra like the one depicted in the inset of Fig. 6c^47^. Indeed, after allowing for a Gaussian broadening, the experimental data can be fitted using a symmetric superposition of two double-horn contributions (Fig. 6c) [the additional sub-structure of the narrow spectral component is related to the sample alignment and therefore not considered further (see “Methods”)].

Given the absence of contrary indications, the magnetic unit cell is taken to coincide with the crystallographic one. There are four translationally-inequivalent P sites, each of which is expected to yield an individual double-horn contribution to the NMR spectrum when a sinusoidal magnetic order with incommensurate variation along *b* is adopted. These sites are related by three space-group reflections, corresponding to mirror and glide planes spanned by the crystal axes^27^. Hence—although it incidentally agrees with previous simulations^5,6^—, the observation of two double-horn contributions with an intensity ratio compatible with 1:1 (see “Methods”) indicates a field-induced breaking of one of these reflection symmetries.

Note that BiCu$_2$PO$_6$ exhibits two types of magnetic layers, which become inequivalent in magnetic fields $\varvec{H} \parallel b$ [black arrows in Fig. 2a]. This provides a natural explanation for the observed NMR line shape. At the level of the spin-Hamiltonian governing electronic and nuclear magnetic moments, this scenario corresponds to a significantly nonlinear coupling with the external magnetic field. Even a coupling between the magnetic field and the longitudinal chirality of the magnetic moments is conceivable. However, in this case, the experimentally-observed coincidence of the centers of the two double-horn contributions would be accidental, which is why we now turn to the scenario of negligible non-Zeeman terms. While the in-plane AFM couplings $J_\perp$ and $J_i$^30,31,37,48^ (Fig. 2a,b) constrain the magnetic structure within each magnetic layer, the coupling between adjacent magnetic layers is weak^37^. The observed NMR spectra can then be explained if residual interlayer couplings give rise to a stacking of magnetically-ordered layers which is not symmetric along the crystallographic *a*-direction. While the significance of quantitative parameter estimates is limited, we find that plausible solutions reproducing the data shown in Fig. 6c *exist* in general within the aforementioned class of spiral magnetic structures (see “Methods”). Furthermore, since the dipole fields created by adjacent magnetic layers decay rapidly with distance along *a*, a randomly-stacked structure in which the propagation phase of adjacent magnetic layers differs by $\pm \delta$ (with $\delta$ fixed) is, in fact, sufficient to explain the results. Thus, the observed NMR spectrum (Fig. 6c) is fully consistent with a spiral magnetic structure, as it is expected to emerge due to field-induced chirality in the frustrated-ladder model in the vicinity of interladder couplings.

An electric polarization oriented predominantly along *a* has been reported to appear at $H_{c2}$ in an order-parameter-like manner^42^. Indeed, magnets exhibiting spiral order often are improper ferroelectrics^49^. In BiCu$_2$PO$_6$, magnetic fields $\varvec{H} \parallel b$ preserve a two-fold screw-axis symmetry, which the magnetic order must break in order to induce a polarization perpendicular to *b* [see section 8.6.5 of Ref. 50 for an extended discussion]. This corroborates the aforementioned reduced-symmetry stacking of magnetic layers.

Considering the NMR spectra in Fig. 6, we notice increasing distortions upon approaching the $H_{c2}$ phase boundary from higher fields or lower temperatures, respectively, which indicates the appearance of defects (solitons or domain walls). This is consistent with results of entropy measurements^34^, while data from electric-polarization experiments^42^ suggest that these objects are chiral^51,52^. Their detailed structure or location are hard to infer from NMR data alone, since the hyperfine couplings involve sites with very different propagation phases. Still, the regularity of the distortions implies that defects appear at well-defined locations with respect to the magnetic structure. Such localization effects can be due to interladder and/or magneto-elastic^38^ couplings, as in CuGeO$_3$^53–57^.

While the numerical predictions of a field-induced chiral phase are consistent with the experimental data, previous numerical signs^5,6^ of solitonic behavior could not be substantiated. We interpret this as a pointer to the importance of interactions beyond the one-dimensional frustrated-ladder model at magnetic fields $H \lesssim H_{c2}$ (cf. preceding paragraph). Since DM interactions couple chirality and crystal structure^58,59^, one exciting possibility is that they give rise to a new, DM-based variation of the spin-Peierls instability with associated solitons known^57^ from $\mathrm {CuGeO}_3$. Also, the same unknown interactions may explain the apparent discrepancies between the calculated and the measured^34^ magnetization curves already discussed in Ref. 1 (see also Ref. 50). Future experimental identification of these interactions is crucial to unraveling the precise nature of defects in BiCu$_2$PO$_6$.

To conclude, we performed numerical calculations which enabled us to clarify a part of the in-field phase diagram of the frustrated spin ladder model and presented experimental evidence that the field-induced phase observed in BiCu$_2$PO$_6$ for $H > H_{c2}$ ($\varvec{H} \parallel b$) has a spiral magnetic structure. Our numerical results show that this order is driven by the field-induced chirality in the individual one-dimensional ladder units, whereas the presence of spiraling ordered moments is a mere secondary effect due to the presence of suitable interladder couplings and/or symmetry-lowering DM interactions (see “Methods”). Whilst the frustration within each ladder leg is essential for the field-induced chirality, the rung bonds of the ladder support this effect by weakening the apparently competing dimer order. Furthermore, our experiments indicate that the behavior of BiCu$_2$PO$_6$ at magnetic fields below, or comparable to, $H_{c2}$ is governed by defects not captured by the present one-dimensional model. High-field diffraction experiments could help reveal the nature of such defects, as well as probe the possible magnetic-structure and lattice distortions in the field-induced spiral phase.

## Methods

### Numerical calculations

The Hamiltonian (1) is studied using exact diagonalization and density-matrix renormalization group^60^ (DMRG). Following previous work^5,6^, we consider individual spin operators $\varvec{S}_i$, the dimer operator $\varvec{S}_i \cdot \varvec{S}_j$, as well as the chirality $\varvec{\kappa }_{ij}$, and compute their correlation functions for individual ladders. The field-induced phases are identified by inspecting the aforementioned correlation functions (see, e.g., Ref. 20) and the associated structure factors (cf. Ref. 6). The correlation functions are averaged over several reference sites/bonds close to the center of the ladder^61^. The convergence^62^ of the results as a function of the matrix bond dimension and the number of optimization sweeps was checked. Moreover, the ground-state degeneracy was accounted for. Finite-size effects^61^ were checked by performing calculations for several system sizes. The abscissa range shown in Fig. 3 was restricted to exclude regions strongly affected by the open system boundaries.

The symmetric anisotropy tensor $\varvec{\Gamma }_{ij}$ is given by^44,45^

2 $$\begin{aligned} \varvec{\Gamma }_{ij} = \frac{\varvec{D}_{ij} \otimes \varvec{D}_{ij}}{2\; J_{ij}} - \frac{D_{ij}^2}{4\;J_{ij}}. \end{aligned}$$

Two representative parameter sets—without (*A*) and with (*B*) DM interactions, respectively—are primarily considered in this work:

**Table:** 

Set	$J_1/k_B$	$J_\perp /J_1$	$J_2^\prime /J_1$	$J_2/J_1$	$D_1^{ac}/J_1$	$D_1^b/J_1$
*A*^1^	$140\ \mathrm {K}$	0.75	0.5	1	0	0
*B*^29–31^	$116\ \mathrm {K}$	1	1	1	0.3	0.3

The onset of chiral order and the absence of additional field-induced phases at low magnetization values were checked by simulating systems consisting of up to $L=256$ (128) rungs using bond dimensions of up to $m=2{,}048$ (512) for parameter set *A* (*B*). The results shown in Fig. 3 were obtained using the more recent^29–31^ parameter set *B*. Small differences between chiral correlations at even and odd distances are due to the staggered DM interactions $D_1^b$ (see Fig. 2c).

In order to make use of the $S_\text {tot}^z$ conservation, most calculations were performed with a fixed g-factor (*g* = 2). Note that the assumption of site-independent g-factors $g_1 = g_2$ is implicit to the parameter set *B*^29–31^. For the parameter set *A*, the inclusion of site-dependent g-factors fitted for $\varvec{H} \parallel b$ ($g_1 = 1.78$, $g_2 = 2.19$) did not affect any of the reported conclusions. The data shown in Fig. 5 were obtained by varying the parameter set *A* as indicated in the figure. Here, the uncertainties in the phase boundaries are due to practical limitations (except for parameter set *A*, $L=64$ and $m=512$ were used). Taking $\Delta {}J_F = J_2 - J_2^\prime \rightarrow 0$ for parameter set *A* [while keeping $J_F =(J_2^\prime +J_2)/2$ constant] did not affect the field-induced phases below half-saturation magnetization and the results obtained for $J_\perp = 0$ were consistent with those reported in Ref. 20. Moreover, the zero-field ground states were consistent with those reported^11^ for $\Delta {}J_F = 0$. Hence, the comparison of our results with previous results^11^ obtained for the case $J_2^\prime =J_2$ is justified.

To study the effect of DM interactions, we augmented the parameter set *A* by individual symmetry-allowed DM terms ($D_1^b = 0.2\,J_1$, $D_1^{ac} = 0.35\,J_1$, $D_4^b = 0.2\,J_1$, ${D^\prime }_2^a \approx D_2^a = 0.4\,J_1$, and ${D^\prime }_2^c \approx D_2^c = 0.4\,J_1$; cf. Refs. 29,50). For historical reasons and following standard practice^63–67^, we set $\varvec{\Gamma }_{ij}=0$ in these calculations. In all the considered cases, the field-induced chiral phase persisted up to magnetizations of at least $\sim 20\%$ of the saturation magnetization. [The interplay between transverse DM interactions and transverse chirality correlations corresponding to spin canting tends to pin the spiral phase in finite-size systems, resulting in the appearance of spiraling ordered moments in the DMRG calculations (section 7.14.2 of Ref. 50, as well as previous work^5,6^). This is believed to be forbidden by symmetry in the infinitely-extended system (cf. “Introduction”) and we have checked that the data shown in Fig. 3 are consistent with results obtained for another system size ($L=64$). Nevertheless, the described effect may have implications for the long-range magnetic ordering in large-but-finite crystals.]

Additional results and details are documented in Ref. 50.

### High-field NMR: experimental details

The NMR shifts are reported relative to a standard $^{31}$P reference^68^. The total uncertainty in the field calibration is estimated as 0 to $150\ \mathrm {ppm}$ (mostly correlated and systematic), with a tendency to overestimate the magnetic field at the sample position. The spin-lattice relaxation rate $T_1^{-1}$ was obtained by fitting a stretched-exponential recovery, with stretching exponents ranging from 0.6 to 1. The single-crystalline BiCu$_2$PO$_6$ sample^69^ was mounted on an NMR probe featuring a two-axis rotator. After pre-alignment in a superconducting magnet at $15\ \mathrm {T}$ (using the angular dependence^70^ of the $^{31}$P-NMR shift), the sample was realigned in-situ in the high-field magnet. Differences with respect to Ref. 5 are attributed to the lack of such an alignment facility in the previous experiments. Nevertheless, a small residual misalignment remained due to technical limitations. In fact, the fine structure of the NMR spectra shown in Fig. 6 was found to disappear upon a subtle change of sample orientation (checked at $\mu _0\,H=37.7\ \mathrm {T}$). The low-frequency shoulder visible in the dark-blue data plotted in Fig. 6a appeared to be affected by the sample orientation as well, which suggests an experimental origin. Additional $^{31}$P-NMR and magnetization measurements performed after the high-field experiments confirmed the sample integrity.

### High-field NMR: relative intensities

The relative intensity of the two double-horn components fitted to the data in Fig. 6c is approximately 3:2. Yet, the group of reflections linking the four translationally-inequivalent $^{31}$P sites (see main text) is isomorphic to $Z_2 \times Z_2$. Therefore, essential degeneracies can only give rise to one, two, or four double-horn contributions with *equal* intensities. There are two possible causes for the apparently reduced amplitude of the narrow component of the spectrum. First, the nuclear-spin dynamics are most likely non-uniform across the NMR spectrum, as evidenced by the fact that a comb of 50 saturation pulses fully suppressed the signal at frequencies close to the edge of the spectrum, while failing to do so for frequencies near the center of the line (at $\mu _0{}\,H = 42.2\ \mathrm {T}$, $T = 1.7(2)\ \mathrm {K}$; nuclear polarization probed after $30\ \mathrm{ms}\ll T_1$). Second, the NMR spectra were obtained by summing Fourier-transformed spin-echoes recorded at different frequencies^71^. Since signals were strong in general, non-linear response of the receiving electronics could have led to reduced intensities of the central peak.

### Coupling parameters and uncertainties

The hyperfine field $\varvec{B}_\text {hf}$ at each $^{31}$P nucleus is written as $\varvec{B}_\text {hf} = \sum _i (A_i + D_i)\, \varvec{\mu }_i$, with the sum running over all electronic magnetic moments $\varvec{\mu }_i = -\mu _{\mathrm {B}}\,g_i\,\langle \varvec{S}_i \rangle$. After fixing the exchange couplings $(J_1,J_2^\prime ,J_2,J_\perp )$ proposed in Ref. 1, the g-tensors $g_i$ of the two magnetic sites were obtained by fitting the results of full-spectrum exact-diagonalization calculations to the low-field magnetization data from Ref. 50,70. Approximate $C_{2v}$-symmetry^70^ of the CuO$_4$ plaquettes^27^ is assumed. We further assume that the g-tensors are symmetric and positive. It turns out that only one of the two approximate $C_{2v}$-symmetry–axes differs significantly between the two Cu sites. Subsequently, the anisotropic hyperfine couplings $A_i$, linking^28^ each P nucleus to four surrounding magnetic sites (Fig. 2b), can be estimated from the angular dependence^70^ of the NMR shift in the paramagnetic state of pristine and slightly Zn-doped BiCu$_2$PO$_6$^50, 70^. We assume that the corresponding matrices are symmetric^70^. The dipole couplings $D_i$ are treated by means of a plane-wise summation technique^72,73^, using the crystal structure reported in Ref. 27. By repeating the analysis with $J_2^\prime = J_2 = 0.75\,J_1$, we estimate uncertainties up to the order of $25\%$ for the elements of the matrices $A_i\,g_i$, and $10\%$ for those of $g_i$^50^. Note, however, that more pessimistic considerations suggest errors of order $100\%$ and more^50^. The details of the above analyses are beyond the scope of this work and have been documented in Refs. 50,70.

### Models for the NMR line shape

The NMR spectrum expected for any hypothetical magnetic structure can be calculated if the following properties are known: (i) the g-tensors for both magnetic sites, (ii) the matrices describing the hyperfine couplings, and (iii) the crystal structure. While the former two can be estimated from low-field data, the resulting parameters have large uncertainties (see preceding section). Moreover, a magnetic field lowers the crystal symmetry (see main text), such that field-induced lattice deformations could in principle alter any of the three aforementioned properties. In addition, the quantitative information contained in the NMR spectrum (Fig. 6c)—i.e., the widths and the center frequency of the two double-horn components—is insufficient to constrain even the simplest spiral models. Despite these limitations, quantitative analyses of the NMR line shape were attempted and shall be briefly described below.

Following the main text, a spiral magnetic structure analogous to that shown in Fig. 4 is assumed to form within each magnetic layer of BiCu$_2$PO$_6$. The propagation wavenumber along *b* is fixed to ${q_b = 0.574}$^30,31,37^. We also include a tilt of the spiral axis, as it is expected to occur due to the recently-suggested^30,31^ DM interaction $D_1^{ac}$. The twist-distortion associated with the also suggested^30,31^ DM interaction $D_1^b$ is not considered—in order to limit the number of degrees of freedom and because numerical calculations suggest that the associated angles are smaller^50^. Since the longitudinal magnetization determines only the NMR shift, but not the NMR line shape, we end up with five parameters (the wavenumber $q_a$ describing the propagation of the magnetic structure along *a*, two tilt angles, and two transverse ordered moments $m_{\perp ,i} \le 0.5\,\hslash$), which are adjusted in order to reproduce the widths of the two observed double-horn contributions shown in Fig. 6c. To account for the aforementioned uncertainties, we consider the corresponding one- and two-parameter variations of the coupling parameters. For each resulting set of coupling parameters, candidate solutions reproducing the experimental data exist. Furthermore, the aforementioned model has been generalized to include random stacking configurations of magnetically-ordered layers. In particular, taking a candidate solution with $q_a = 0.5$ and recomputing the line shape assuming a random stacking scenario with co-aligned chirality and phase shift $\delta = \pm \pi /2$ (see main text) yielded only small changes to the spectrum. For additional information, as well as detailed symmetry considerations covering more general spiral magnetic structures, we refer to Ref. 50.
