# Publisher Correction: Two coupled chains are simpler than one: field-induced chirality in a frustrated spin ladder

**DOI:** 10.1038/s41598-021-82922-w

**Published:** 2021-02-03

**Authors:** Marek Pikulski, Toni Shiroka, Francesco Casola, Arneil P. Reyes, Philip L. Kuhns, Shuang Wang, Hans-Rudolf Ott, Joël Mesot

**Affiliations:** 1 Laboratory for Solid State Physics, ETH Zürich, 8093 Zürich, Switzerland; 2 Paul Scherrer Institut, Villigen PSI, 5232 Villigen, Switzerland; 3 Harvard-Smithsonian Center for Astrophysics, Harvard University, Cambridge, MA 02138 USA; 4 National High Magnetic Field Laboratory, Florida State University, Tallahassee, FL 32310 USA; 5 Laboratory for Quantum Magnetism, Ecole Polytechnique Fédérale de Lausanne, 1015 Lausanne, Switzerland

Correction to: *Scientific Reports* https://doi.org/10.1038/s41598-020-72215-z, published online 28 September 2020

This Article contains a typographical error in the Methods section under subheading ‘Coupling parameters and uncertainties’, where

“After fixing the exchange couplings $(J_1,J_2^\prime ,J_2,J_\perp)$ proposed in Ref. 1, the g-tensors $g_i$ of the two magnetic sites were obtained by fitting the results of full-spectrum exact-diagonalization calculations to the low-field magnetization data from Ref. 50,70.“

should read:

“After fixing the exchange couplings $(J_1,J_2^\prime ,J_2,J_\perp)$ proposed in Ref. 1, the g-tensors $g_i$ of the two magnetic sites were obtained^50^ by fitting the results of full-spectrum exact-diagonalization calculations to the low-field magnetization data from Ref. 70.”

